# Functional Connectivity and Compensation of Phonemic Fluency in Aging

**DOI:** 10.3389/fnagi.2021.644611

**Published:** 2021-07-05

**Authors:** Rosaleena Mohanty, Lissett Gonzalez-Burgos, Lucio Diaz-Flores, J-Sebastian Muehlboeck, José Barroso, Daniel Ferreira, Eric Westman

**Affiliations:** ^1^Division of Clinical Geriatrics, Center for Alzheimer Research, Department of Neurobiology, Care Sciences, and Society, Karolinska Institutet, Stockholm, Sweden; ^2^Department of Clinical Psychology, Psychobiology and Methodology, Faculty of Psychology, University of La Laguna, San Cristóbal de La Laguna, Spain; ^3^Hospital Universitario de Canarias, San Cristóbal de La Laguna, Spain; ^4^Department of Radiology, Mayo Clinic, Rochester, MN, United States; ^5^Department of Neuroimaging, Centre for Neuroimaging Sciences, Institute of Psychiatry, Psychology and Neuroscience, King's College London, London, United Kingdom

**Keywords:** resting-state, functional MRI, functional connectivity, verbal fluency, phonemic fluency, cognitive reserve, compensation, healthy aging

## Abstract

Neural compensatory mechanisms associated with broad cognitive abilities have been studied. However, those associated with specific cognitive subdomains (e.g., verbal fluency) remain to be investigated in healthy aging. Here, we delineate: (a) neural substrates of verbal (phonemic) fluency, and (b) compensatory mechanisms mediating the association between these neural substrates and phonemic fluency. We analyzed resting-state functional magnetic resonance imaging from 133 right-handed, cognitively normal individuals who underwent the Controlled Oral Word Association Test (COWAT) to record their phonemic fluency. We evaluated functional connectivity in an established and extended language network comprising Wernicke, Broca, thalamic and anti-correlated modules. (a) We conducted voxel-wise multiple linear regression to identify the brain areas associated with phonemic fluency. (b) We used mediation effects of cognitive reserve, measured by the Wechsler Adult Intelligence Scale—Information subtest, upon the association between functional connectivity and phonemic fluency tested to investigate compensation. We found that: (a) Greater functional connectivity between the Wernicke module and brain areas within the anti-correlated module was associated with better performance in phonemic fluency, (b) Cognitive reserve was an unlikely mediator in younger adults. In contrast, cognitive reserve was a partial mediator of the association between functional connectivity and phonemic fluency in older adults, likely representing compensation to counter the effect of aging. We conclude that in healthy aging, higher performance in phonemic fluency at older ages could be attributed to greater functional connectivity partially facilitated by higher cognitive reserve, presumably reflecting compensatory mechanisms to minimize the effect of aging.

## Introduction

Despite changes to brain integrity with aging, some functions such as language processes remain remarkably preserved (Schaie and Willis, 1993; Ansado et al., 2013). One theory for the maintenance of language function in light of age-related brain atrophy is the engagement of compensatory brain networks (Park and Reuter-Lorenz, 2009). The impact of age-related atrophy on various cognitive domains is well-documented (Raz and Rodrigue, 2006; Ferreira et al., 2014; Lowe et al., 2019; Sungura et al., 2020). Hence, investigating how compensatory mechanisms in specific cognitive domains (e.g., language) counteract the onslaught of aging may articulate ways to improve everyday functioning of older adults.

Traditionally, the characterization of the language network in the brain has been limited to the Broca's area (inferior frontal) and Wernicke's area (superior temporal), which are associated with the functions of language production and language comprehension, respectively (Mesulam, 1998). However, recent resting-state functional connectivity and lesion studies have implicated the contribution of a more extended network in language processing (Costafreda et al., 2006; Birn et al., 2010; Tomasi and Volkow, 2012; Zhang et al., 2013; Marsolais et al., 2014, 2015; Methqal et al., 2019). In the elderly, decline in language functions such as experiencing difficulty in retrieval of words is common and could be an early indicator of presence of pathology (Henry and Crawford, 2004). Such deficits can be captured by cognitive tests evaluating verbal fluency, particularly sensitive toward frontal lobe functions, critical for retrieval, free recall and executive functions (Jurado et al., 2000; Azuma, 2004; Henry and Crawford, 2004; Cabeza and Dennis, 2012; Robinson et al., 2012).

One of the subdomains of verbal fluency is phonemic fluency, which is commonly used in clinical practice and research. Phonemic fluency tests require participants to say as many words as possible beginning with a specific letter (e.g., F, E) usually within 1 min. Phonemic fluency has been found to be relatively stable during aging in comparison to other fluency domains (Troyer, 2000; Foldi et al., 2003; Elgamal et al., 2011; Gonzalez-Burgos et al., 2019). Although verbal fluency is typically measured based on the number of timely acceptable answers, alternative qualitative measures have been proposed, including clustering (groupings or contiguous words in the same sub-category) and switching (changing groupings) to infer the nature of the deficit (Troyer et al., 1997; Troyer, 2000). Impaired clustering reflects compromised temporal cortex, while impaired switching reflects compromised dorsolateral and superior medial frontal regions (Troyer et al., 1998). A fall in switching performance is evident in advanced ages (Troyer et al., 1997; Pereira et al., 2018) and is associated with impaired working memory. In contrast, the stability in phonemic fluency performance has been linked with the contribution of other cognitive domains (Troyer et al., 1998), including a more efficient use of ipsilateral language networks (Gonzalez-Burgos et al., 2020), and perhaps involving a greater capacity to recruit contralateral frontoparietal networks (Gonzalez-Burgos et al., 2019). Resting-state MRI-based functional connectivity studies have identified four modules related to performance in phonemic fluency (Tomasi and Volkow, 2012), including the Broca module, Wernicke module, thalamic module and anti-correlated module.

Although not entirely characterized, ipsilateral language networks and contralateral frontoparietal networks may form the basis for compensation and the stability in phonemic fluency, which could be facilitated by higher levels of cognitive reserve (Gonzalez-Burgos et al., 2020). Cognitive reserve is “the adaptability of cognitive processes that helps to explain differential susceptibility of cognitive abilities or day-to-day function to brain aging, pathology, or insult” (Stern et al., 2018a, 2020). Individuals with higher cognitive reserve produce more words in phonemic fluency (Crossley et al., 1997; Tombaugh et al., 1999; Auriacombe et al., 2001; Roldán-Tapia et al., 2012; Balduino et al., 2020), have greater neural efficiency (Bartrés-Faz et al., 2009; Fernández-Cabello et al., 2016), and thus, have greater potential for compensation. The closest direct measure of cognitive reserve is based on functional brain processes (Stern et al., 2020), addressed with methods such as functional magnetic resonance imaging (MRI). Despite the wealth of research on identifying and characterizing distinct aspects of verbal fluency, limited neuroimaging studies have investigated the neural correlates of phonemic fluency, specifically in the context of cognitive reserve (Boyle et al., 2020; Rodríguez-Aranda et al., 2020).

To this end, we aimed to study the neural functional substrates of phonemic fluency and potential compensatory mechanisms facilitated by cognitive reserve, which would contribute to high performance in phonemic fluency across age groups. Firstly, we characterized resting-state functional MRI-based functional connectivity within the four brain modules previously related with phonemic fluency (Tomasi and Volkow, 2012). By including these four modules, we extended beyond the traditional Broca's and Wernicke's brain areas and tested for both within and outside network effects, which may be relevant for investigating compensation. We described functional connectivity patterns in younger and older adults as well as tested for potential differences in functional connectivity between the two age groups. Secondly, we tested for the association between functional connectivity within the four modules and phonemic fluency. Finally, we examined the mediation effect of cognitive reserve on the relationship between functional connectivity and performance in phonemic fluency, separately in younger and older adults. We hypothesized that: (a) younger adults would show greater functional connectivity in the four modules previously related with phonemic fluency than older adults; (b) functional connectivity particularly in Broca's module (language production center) would be associated with performance in phonemic fluency; and (c) cognitive reserve would mediate the relationship between functional connectivity involving both linguistic and non-linguistic brain areas and performance in phonemic fluency, especially in older adults, hence indicating compensation for the effect of age in phonemic fluency.

## Methods

### Participants

A group of 149 cognitively normal healthy individuals were selected from a community-based longitudinal cohort, GENIC (Group of Neuropsychological Studies of the Canary Islands) (Ferreira et al., 2015). For the current study, individuals were selected at the earliest timepoint (not necessarily baseline visit) where a functional MRI was available along with the following inclusion criteria: (a) age ≥35 years; (b) right-handed; (c) normal performance in comprehensive neuropsychological assessment using pertinent clinical normative data (i.e., individuals with mild cognitive impairment or dementia were excluded); (d) preserved global cognitive and functional status operationalized as a Mini-Mental State Examination score (MMSE) ≥24, a Blessed Dementia Scale (BDRS) score <4 and/or a Functional Activity Questionnaire score <6); (e) no neurologic, psychiatric or systemic diseases; (f) no history of substance abuse;(g) no abnormal findings in MRI (e.g. stroke, tumors, hippocampal sclerosis, etc.), as assessed by an experienced neuroradiologist (L.D-F); and (h) a balanced distribution of sex (44.9% female). Although the BDRS scale cut-off for abnormality is frequently established at ≥4 points (Blessed et al., 1968; Erkinjuntti et al., 1988), the “changes in personality, interests and drive” subscale may influence the BDRS total score and does not necessary reflect impairment in activities of daily living (Machado et al., 2018). With the aim of excluding only individuals with functional impairment, as an exception, we included those participants with total BDRS scores ≥4 (*N* = 8) if: (a) 70% or higher percentage of the BDRS total score resulted from “changes in personality, interests and drive” subscale; and (b) if a score ≤ 1.5 was obtained in the other two subscales (“changes in performance of everyday activities” and “changes in habits”). The same procedure has been used in previous studies (Machado et al., 2018; Gonzalez-Burgos et al., 2019). This study was reviewed and approved by the ethics committee of the University of La Laguna, Spain. All participants provided their written and informed consent for participation in this study.

### Neuropsychological Assessments

All individuals underwent an extensive neuropsychological battery, which assessed multiple cognitive domains (language, processing speed, attention, executive functions, episodic memory, procedural memory, visuoconstructive, visuoperceptive and visuospatial functions). To investigate the hypothesis of the current study, we focused on verbal fluency as the primary outcome. Phonemic fluency, a subdomain of verbal fluency, was measured with the Controlled Oral Word Association Test (COWAT) (Benton et al., 1989). Individuals were instructed to recall words beginning with the letters F, A, and S, in a span of 1 min for each letter. Intrusions (proper nouns, numbers, derived words) and perseverations (repetitions of correct words) were considered as errors and were excluded. The total number of correct words was counted and a total score (sum of words for F, A, S) was calculated as the measure of phonemic fluency. Cognitive reserve has been conventionally represented by sociobehavioral proxies (e.g., education, IQ, occupation, etc.). While such proxies may not be able to capture any specific functional mechanisms, they capture experiences contributing toward the development of cognitive reserve. In this study, we chose Wechsler Adult Intelligence Scale-Third Edition (WAIS-III) Information Subtest (Wechsler, 1997), a measure of crystalized intelligence, to represent cognitive reserve. The reasons for this is that the WAIS-III Information subtest has been demonstrated (a) to better capture achievements and usage of educational opportunities compared to years of education (Correia et al., 2015), and (b) to have a greater mediation effect of neural correlates of cognition, when treated as a measure of cognitive reserve compared to other sociobehavioral proxies (WAIS-III Vocabulary, Cognitive Reserve Questionnaire, years of education) (Ferreira et al., 2016), in the current cohort.

### Neuroimaging

All scans were acquired on a 3.0 T GE scanner (General Electric, Milwaukee, WI, USA) with an eight channel high resolution head coil situated at the *Hospital Universitario de Canarias* in Tenerife, Spain. Structural MRI were 3-D T1-weighted fast spoiled gradient echo (FSPGR) scans, acquired sagitally with the following parameters: repetition time (TR) = 8.73 ms, echo time (TE) = 1.74 ms, inversion time (TI) = 650 ms, field of view 250 × 250 mm, matrix 250 × 250 mm, flip angle 12°, slice thickness = 1 mm and voxel resolution = 1 × 1 × 1 mm^3^. Six minutes of resting-state functional MRI were collected using single-shot gradient recalled echo-planar T2^*^-weighted imaging with the following parameters: TR = 2,000 ms, 180 time-points, TE = 22.1 ms, field of view = 240 × 240 mm, flip angle = 90°, matrix = 64 × 64, slice thickness = 4 mm, voxel dimensions 3.75 × 3.75 × 4 mm^3^ and 36 slices on AC-PC orientation. Participants were instructed to relax with their eyes closed while staying awake and head padding were provided to prevent head motion during scanning.

### Functional Connectivity Analyses

Functional MRI were preprocessed with the following steps: the initial six functional volumes were discarded to ensure stabilization of magnetization, the remainder functional volumes were slice time corrected to account for temporal differences in the interleaved acquisition, the volumes were realigned to the mean of all functional volumes for motion correction and to obtain the motion parameters, the mean functional volume was realigned and linearly co-registered to the structural MRI with a rigid body transformation, the structural MRI was segmented into tissue classes (gray matter, white matter and cerebrospinal fluid), the structural MRI was normalized to the standard Montreal Neurological Institute (MNI) space to learn the transformation, the motion-corrected functional MRI volumes were normalized to the MNI space using the learned transformation, and spatially smoothed with 8 mm full width at half maximum Gaussian kernel using Statistical Parametric Mapping software version 12 (https://www.fil.ion.ucl.ac.uk/spm/), all automated through a database system (Muehlboeck et al., 2014). Then, functional MRI were temporally filtered (Gaussian band-pass between 0.01 and 0.1 Hz, implemented with Oxford Center for Functional MRI of the Brain Laboratory Software Library, version 5.0.9, https://fsl.fmrib.ox.ac.uk/fsl/). All scans were assessed visually (raw and registered images) and quality control was based on motion parameters (cases with motion >3 mm or 3° were excluded) and framewise displacement (FWD).

Given the focus on phonemic fluency in this study, we examined functional connectivity in the language functional network, previously identified in healthy individuals (Tomasi and Volkow, 2012). This network comprised 23 regions encompassing four modules: (a) Wernicke module (Wernicke's area plus left middle frontal and superior frontal, bilateral parsorbitalis and inferior temporal, right inferior parietal, pars opercularis and cerebellum); (b) Broca module (Broca's area plus right pars triangularis); (c) thalamic module (thalamus and striatum); and (d) anti-correlated module (anti-correlated to the Wernicke module comprising bilateral visual, auditory and somatosensory brain areas, precuneus and cingulum). The organization and MNI coordinates of the language functional network are presented in Supplementary Figure 1, Supplementary Table 1. For a reasonable trade-off between spatial specificity and temporal signal sensitivity, we represented these regions by defining spheres of radius 6 mm each (Korhonen et al., 2017).

For each module of the language network, the first eigenvariate of the average time course in the involved regions was extracted. Similar time courses were extracted for the white matter and the lateral ventricles. The two latter time courses, the global brain signal changes over time (Li et al., 2019), their derivatives, movement parameters (three translation and three rotation) and corresponding squared values were included as nuisance covariates in the statistical general linear model. Serial correlations were estimated with a restricted maximum likelihood algorithm using an intrinsic autoregressive model during parameter estimation. The effects of interest were tested by linear contrasts, generating statistical parametric T maps in each subject. A contrast image was generated that identified regions significantly correlated to the selected brain region/module after removal of sources of spurious variance at the individual level.

### Statistical Analyses

To address the aims of this study, we divided the data into two age groups, with a threshold at 60 years: a reference control group with younger adults (40–59 years) and a group with the older adults (60–82 years). We conducted both qualitative and quantitative analyses to examine group characteristics, group differences, associations with phonemic fluency and cognitive reserve mediations potentially reflecting compensatory mechanisms. All the analyses were performed using MATLAB R2014b (The MathWorks, Inc., Natick, Massachusetts, United States).

#### Mean Functional Connectivity in Younger and Older Adults

Functional connectivity patterns specific to each module and age group were identified through voxelwise one-sample *t*-test, revealing brain regions with functional connectivity greater than the global mean value. Multiple comparisons correction was performed with Family-wise Error (FWE) *p* ≤ 0.001. Sex was included as a potential covariate and the whole procedure was repeated for each of the four modules of the language network.

#### Group Differences in Functional Connectivity Between Younger and Older Adults

To examine the differences in functional connectivity between the younger adults and older adults, we conducted voxelwise two-sample *t*-test at the group-level. The purpose of this analysis was to reveal brain regions showing higher functional connectivity in the younger adults relative to the older adults and vice-versa. Multiple comparisons correction was performed with FWE *p* ≤ 0.05. This whole procedure was repeated for each of the four modules of the language network.

#### Neural Correlates of Phonemic Fluency

We conducted voxelwise multiple linear regression to investigate the association between functional connectivity and phonemic fluency in the combined cohort including both younger and older adults for greater statistical power. The significance of the obtained clusters was based on whether they achieved an extent threshold corresponding to a whole-brain FWE-corrected *p* ≤ 0.05 and a cluster-forming threshold of uncorrected *p* ≤ 0.001. Given that such a parametric clusterwise inference approach may inflate the false positive rate based on the assumptions of Gaussian random field theory (Eklund et al., 2016), we verified the association between functional connectivity and phonemic fluency with a non-parametric approach (SnPM13.1.08), which is free of such assumptions (Nichols and Holmes, 2002). Here, permutation testing (5,000 permutations) determined the significance of the obtained clusters that achieved an extent threshold corresponding to FWE-corrected *p* ≤ 0.05 and a cluster-forming threshold of FWE-corrected *p* ≤ 0.05. Finally, we reported the clusters achieving significance in parametric and validated by non-parametric clusterwise inference approaches. Cluster-level functional connectivity was computed as the first eigenvariate over the whole significant cluster which was used for subsequent *post-hoc* analyses. We accounted for the timepoint of the visit (baseline vs. follow-up) to control for potential learning effects in phonemic fluency. Additionally, to understand the contribution of language function to phonemic fluency, we controlled for other cognitive functions which may also underlie phonemic fluency in independent parametric models. Specifically, we controlled for mental tracking and cognitive flexibility/executive control (assessed by time for completion of the Color Trails Test—Part 2), verbal memory (assessed by learning across three trials in TAVEC: *Test de Aprendizaje Verbal España-Complutense*) or processing speed (assessed by reaction time in Vienna Reaction test) (Ferreira et al., 2015).

#### Mediation Effects of Cognitive Reserve

We examined potential mediation effects of cognitive reserve on the association between functional connectivity and phonemic fluency. We first conducted a mediation analysis in the combined cohort, irrespective of age, to understand the relationship of cognitive reserve with cluster-level functional connectivity and phonemic fluency. Once demonstrated that cognitive reserve can modify the association between functional connectivity and phonemic fluency, we investigated whether this effect may be more prominent in older individuals, thus, delineating compensatory effects. In particular, we tested the direct involvement of functional connectivity as well as the indirect involvement of functional connectivity mediated by cognitive reserve (WAIS-III Information subtest) in relation to phonemic fluency (Baron and Kenny, 1986). We implemented the mediation model through a series of linear regression models comprising four tests. Path *a*: association of functional connectivity with cognitive reserve; path *b*: association of cognitive reserve with phonemic fluency; direct path *c*: association of functional connectivity with phonemic fluency, and indirect path *c'*: association of functional connectivity and cognitive reserve with phonemic fluency. If associations for paths *a, b*, or *c* are non-significant, then cognitive reserve is an unlikely mediator. If associations for paths *a, b*, and *c* are significant, then path *c'* would indicate either a partial mediation (significant association of functional connectivity with phonemic fluency when controlled for cognitive reserve) or a full mediation (non-significant association of functional connectivity with phonemic fluency when controlled for cognitive reserve) effect of cognitive reserve.

## Results

### Participants

From the initial cohort of 149 individuals, data from two individuals were excluded after initial quality control of the scans (due to short scan length, limited FOV). Based on the motion parameters, data from fourteen (older) individuals were excluded (mean FWD > 0.25 mm). Table 1 shows the demographic and clinical characteristics of the remainder 133 individuals included for further analyses. There were no significant differences in the sex distribution (*p* = 1), WAIS-III Information subtest (*p* = 0.5), and FWD (*p* = 0.07) between the younger adults (40–59 age years) and older adults (60–82 years). However, there were significant differences between the two groups in MMSE (*p* < 0.001), and phonemic fluency (*p* = 0.003; *p* = 0.06 when controlled for MMSE).

**Table 1:** Demographics and clinical characteristics of the study cohort.

	**Combined cohort (*N* = 133)**	**Younger adults(*N* = 60)**	**Older adults (*N* = 73)**	***p*-value**
Baseline (BL) vs. follow-up (FU) visit (BL/FU)	23/110	3/57	20/53	-
Age (years)	60.7 ± 9.3 (40, 82)	52.1 ± 4.6(40, 59)	67.7 ± 5.5 (60, 82)	** <0.0001**
Sex (%F)	43.6	43.3	43.8	1
MMSE	29.4 ± 1.1 (25, 30)	29.8 ± 0.6(27, 30)	29.1 ± 1.3 (25, 30)	** <0.001**
Phonemic fluency (words)	36.4 ± 12.8 (11, 72)	39.9 ± 10.8(16 69)	33.5 ± 13.7 (11, 72)	**0.003**
WAIS-III Information	17.1 ± 5.8(5, 27)	17.4 ± 5.2(7, 25)	16.7 ± 6.3(5, 27)	0.5
FWD (mm)	0.07 ± 0.02 (0.03, 0.16)	0.07 ± 0.03(0.03, 0.16)	0.07 ± 0.02 (0.03, 0.13)	0.07

*N, sample size; F, female; MMSE, mini mental state examination; WAIS-III, Wechsler Adult Intelligence Scale; FWD, framewise displacement. All continuous variables are reported as mean ± standard deviation (range). Group differences between younger adults and older adults were assessed using hypothesis testing with two sample t-test for continuous variables and Fisher exact test for categorical variables. In bold, p ≤ 0.05 for easier discrimination*.

### Mean Functional Connectivity in Younger and Older Adults

**Figure 1:**
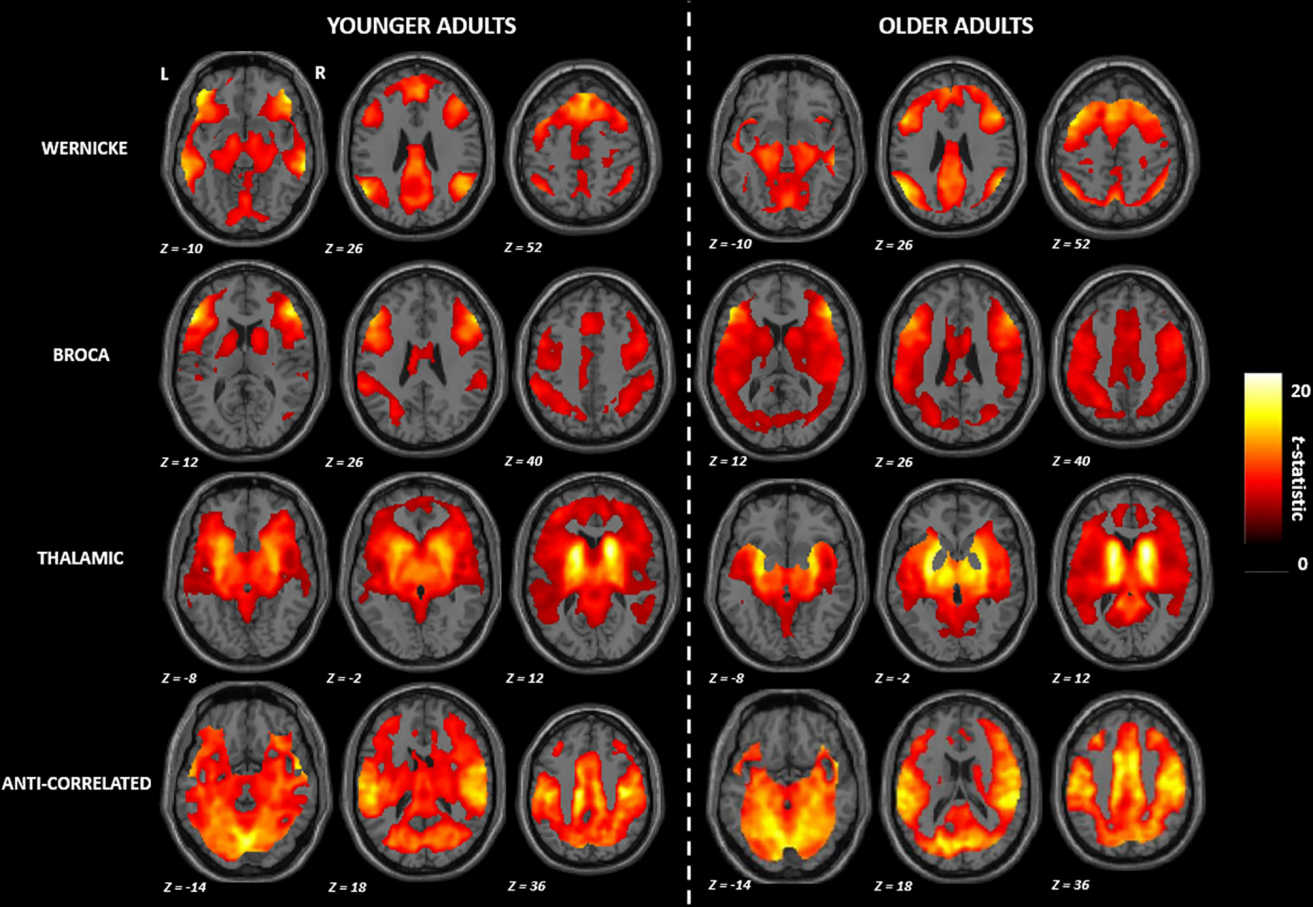
Group mean maps observed in the functional connectivity of the language network. R, right; L, left. All brain maps are visualized at familywise error corrected *p* ≤ 0.001.

Figure 1 shows the mean functional connectivity maps (FWE corrected *p* ≤ 0.001) for each module of the language network by age groups. Based on qualitative visual inspection, two patterns emerged: (a) in the Wernicke and the Broca modules, the younger adults demonstrated a distinct pattern of functional connectivity localized to specific brain areas while the older adults demonstrated a diffuse pattern of functional connectivity involving additional brain regions; (b) in the thalamic and the anti-correlated modules, the younger adults demonstrated involvement of greater number of brain regions relative to the older adults. All of these differences persisted after adjusting for sex as a potential covariate.

### Group Differences in Functional Connectivity Between Younger and Older Adults

**Figure 2:**
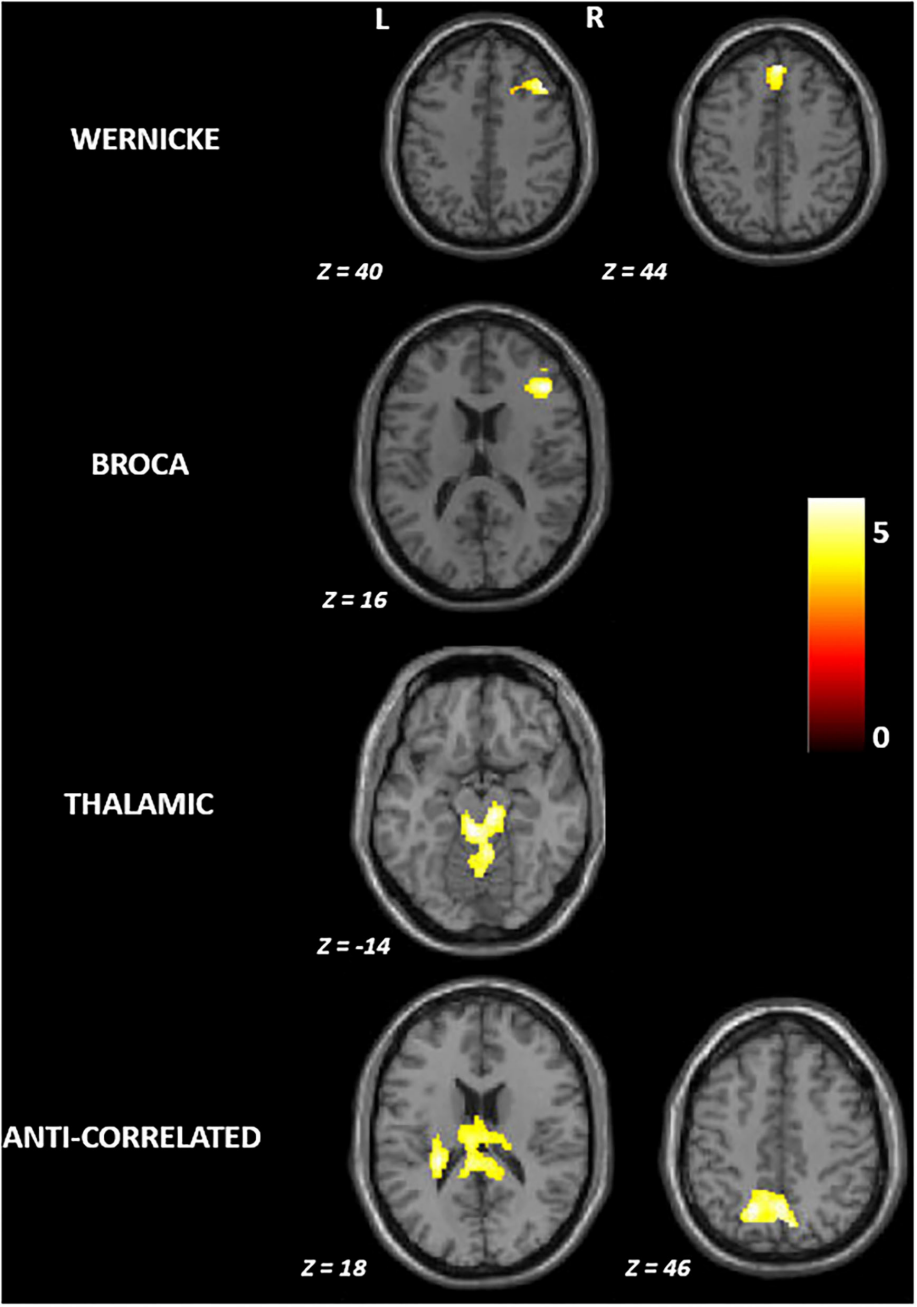
Group differences in functional connectivity between younger and older adults in the language network. R, right; L, left. All brain maps are visualized at familywise error corrected *p* ≤ 0.05; The colorbar represents *t*-statistic.

Figure 2 and Table 2 summarize the findings on differences in functional connectivity between the younger and the older adults. In all four modules, we observed higher functional connectivity in younger than older adults (FWE-corrected *p* ≤ 0.05). Higher functional connectivity was seen in the younger adults involving: (a) the right middle and superior frontal gyrus for the Wernicke module; (b) the right middle frontal gyrus for the Broca module; (c) the brainstem for the thalamic module; and (d) the left parietal operculum and superior parietal lobule for the anti-correlated module. For the contrast testing for brain regions showing higher functional connectivity in older adults than in younger adults, we did not find any significant effects in any module.

**Table 2:** Group differences observed between younger and older adults in functional connectivity of the language network.

**Module**	**Peak region**	**BA**	**MNI coordinates (x, y, z)**	**Z-score**	***p*-value**
Wernicke	R middle frontal gyrus	BA 8	(44, 22, 40)	5.37	0.001
	R superior frontal gyrus	BA 9	(4, 42, 44)	5.25	0.001
Broca	R middle frontal gyrus	BA 46	(44, 32, 16)	4.9	0.007
Thalamic	Brainstem	-	(−4, −34, −14)	4.54	0.032
Anti-correlated	L parietal operculum	BA 41	(−26, −32, 18)	4.84	0.012
	L superior parietal lobule	BA 7	(−16, −60, 46)	4.66	0.025

*R, right; L, left; MNI, Montreal Neurological Institute; BA, Brodmann Area; reported p-values were corrected for multiple comparisons by controlling the familywise error rate*.

### Neural Correlates of Phonemic Fluency

**Figure 3:**
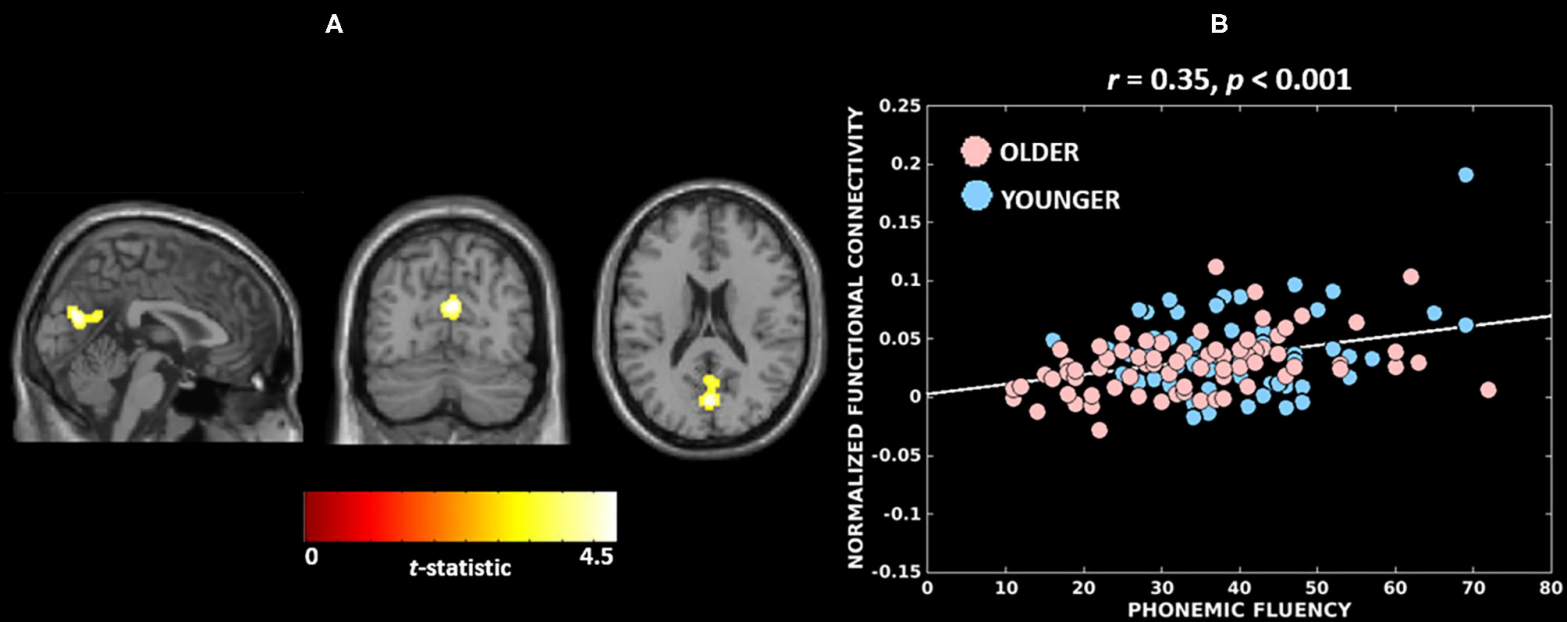
Neural substrates associated with phonemic fluency in the combined cohort. **(A)** A greater functional connectivity of the Wernicke module involving bilateral cuneus (peak) correlates with higher performance in phonemic fluency; The brain maps are visualized at cluster-level familywise error rate corrected *p* ≤ 0.05. **(B)** Association between cluster-level functional connectivity of the Wernicke module and phonemic fluency in younger and older adults.

Figure 3 and Table 3 report the association found between functional connectivity of the Wernicke module and phonemic fluency in the combined cohort. The associated cluster that was deemed significant after parametric (341 voxels, FWE-corrected *p* = 0.033) and further validated with non-parametric (393 voxels, FWE-corrected *p* = 0.030) testing was observed at cuneus (peak at MNI 2, −72, 20). Functional connectivity over the whole cluster, computed *post-hoc* as the first eigenvariate, accounted for 82.8% of the variance. Specifically, greater functional connectivity of the Wernicke module involving ipsilateral and contralateral cuneus and extending to precuneus, calcarine cortex and lingual gyrus (Figure 3A), was significantly associated with higher phonemic fluency at the cluster-level (*r* = 0.35, *p* < 0.001). Notably, older adults with lower functional connectivity presented poorer phonemic fluency compared to the younger adults (Figure 3B). When evaluating the effect of non-language cognitive functions towards phonemic fluency, we observed from the parametric models that the association between functional connectivity of the Wernicke module and phonemic fluency was not significant when controlled for time for completion of the Color Trails Test—Part 2 (N=126; FWE corrected *p*-values: peak-level *p* = 0.2; cluster-level *p* = 0.4; peak region = contralateral and ipsilateral cuneus) but it was significant when controlled for learning in TAVEC (*N* = 124; FWE corrected *p*-values: peak-level *p* = 0.021; cluster-level *p* = 0.019; peak region = contralateral and ipsilateral cuneus) and the reaction time in Vienna Reaction test (*N* = 116; FWE corrected *p*-values: peak-level *p* = 0.015; cluster-level *p* = 0.001; peak region = contralateral and ipsilateral cuneus). We did not observe any significant associations between functional connectivity and phonemic fluency for the Broca, thalamic or anti-correlated modules.

**Table 3:** Correlates and mediator of phonemic fluency.

**Language module: Wernicke**			
**Functional connectivity correlates**			
Peak region			Contralateral and ipsilateral cuneus
MNI coordinates for peak region (x, y, z)			(2, −72, 20)
Parametric cluster size (voxels)			341
Parametric FWE corrected *p*-value			0.033
Non-parametric cluster size (voxels)			393
Non-parametric FWE corrected *p*-value			0.03
*r* (*p*-value)			0.35 (<0.001)
**Cognitive reserve as a mediator**			
**Coefficient for path**	**Combined cohort**	**Younger adults**	**Older adults**
*a* (*p*-value)	2.2 (0.03)	0.2 (0.8)	2.9 (0.005)
*b* (*p*-value)	7.4 (<0.001)	2.7 (0.01)	7.4 (<0.001)
*c* (*p*-value)	4.4 (<0.001)	1.9 (0.06)	3.8 (<0.001)
*c'* (*p*-value)	3.7 (0.003)	1.9 (0.06)	2.4 (0.02)

*MNI, Montreal Neurological Institute; FWE, familywise error; r, correlation coefficient; a, corresponds to the model testing association between cluster-level functional connectivity and cognitive reserve; b, corresponds to the model testing association between cognitive reserve and phonemic fluency; c, corresponds to the model testing association between cluster-level functional connectivity and phonemic fluency (direct path); c', corresponds to the model testing association between cluster-level functional connectivity, cognitive reserve together and phonemic fluency (indirect path)*.

### Mediation Effects of Cognitive Reserve

**Figure 4:**
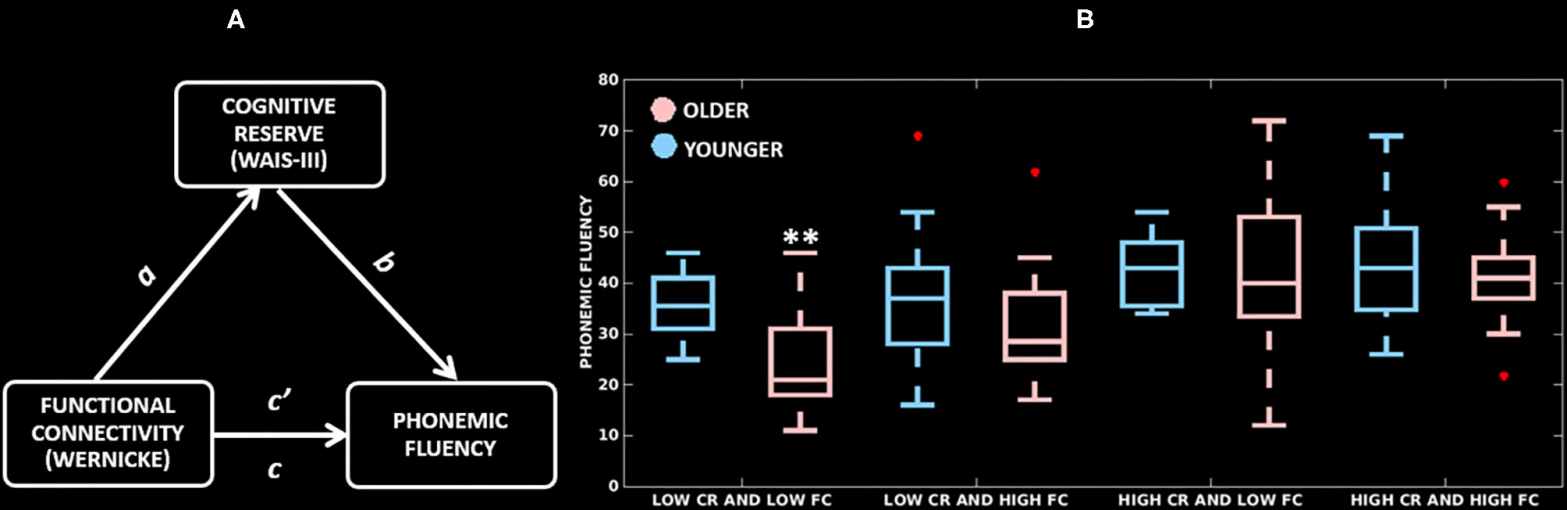
Mediation effects of cognitive reserve. **(A)** Mediation model testing cognitive reserve as a mediator between cluster-level functional connectivity of the Wernicke module and phonemic fluency; *a*: association of functional connectivity with cognitive reserve; *b*: association of cognitive reserve with phonemic fluency; direct path *c'*: association of functional connectivity with phonemic fluency, and indirect path *c*: association of functional connectivity and cognitive reserve with phonemic fluency. **(B)** Distribution of cluster-level functional connectivity of the Wernicke module (independent variable, two groups based on the median-split), cognitive reserve (mediator, two groups based on the median-split) and phonemic fluency (dependent variable). CR, cognitive reserve; FC, functional connectivity; **significantly lower phonemic fluency compared to all other groups (*p* ≤ 0.01).

First, we wanted to demonstrate that cognitive reserve mediates the association between functional connectivity of the Wernicke module and performance in phonemic fluency. This analysis was performed in the combined cohort (younger and older groups pooled together). We did not run any model for the Broca, thalamic and anti-correlated modules because functional connectivity in these modules did not correlate with performance in phonemic fluency. Figure 4A and Table 3 present the mediation model for the Wernicke module within the significant cluster that correlated with phonemic fluency. In the combined cohort, we found a significant association between: greater functional connectivity of the Wernicke module and higher cognitive reserve (coefficient *a* = 2.2, *p* = 0.03); higher cognitive reserve and higher performance in phonemic fluency (coefficient *b* = 7.4, *p* < 0.001); greater functional connectivity of the Wernicke module and higher performance in phonemic fluency (direct path coefficient *c* = 4.4, *p* < 0.001); greater functional connectivity of the Wernicke module and higher performance in phonemic fluency when controlled for cognitive reserve (indirect path coefficient *c'* = 3.7, *p* = 0.003). Thus, cognitive reserve was found to be a partial mediator between functional connectivity of the Wernicke module and phonemic fluency in the combined cohort.

Once demonstrated that cognitive reserve mediates the association between the cluster-level functional connectivity and phonemic fluency, we wanted to investigate whether this effect is differential in younger and older adults (Table 3), with a greater mediation effect in older adults, hence delineating compensation. In the younger adults, there was no significant association between functional connectivity of the Wernicke module and cognitive reserve (coefficient *a* = 0.2, *p* = 0.8). This non-significant result indicated that the mediation effect of cognitive reserve is unlikely in the younger adults. Contrarily, in older adults, there was a significant association between: greater functional connectivity of the Wernicke module and higher cognitive reserve (coefficient *a* = 2.9, *p* = 0.005); higher cognitive reserve and higher performance in phonemic fluency (coefficient *b* = 7.4, *p* < 0.001); and greater functional connectivity of the Wernicke module and higher performance in phonemic fluency (direct path coefficient *c* = 3.8, *p* < 0.001). Finally, the association between functional connectivity of the Wernicke module and phonemic fluency, when controlled for cognitive reserve was significant (indirect path coefficient *c'* = 2.4, *p* = 0.02). This result indicates that cognitive reserve was a partial mediator between functional connectivity of the Wernicke module and phonemic fluency in older adults.

Figure 4B highlights the mediation effect of cognitive reserve levels on the association between the cluster-level functional connectivity of the Wernicke module and phonemic fluency. Across the low/high (median-split) cognitive reserve and low/high (median-split) functional connectivity groups, (a) older adults with low cognitive reserve and low functional connectivity showed the poorest phonemic fluency compared to all of the other groups (*p* ≤ 0.01), but (b) older adults with high cognitive reserve showed phonemic fluency comparable to their younger counterparts, indicating ability to compensate for the effect of aging in phonemic fluency.

## Discussion

Cognitive status is instrumental in maintenance of functional independence in the elderly. Decline in cognitive status could arise due to age-related pathology—manifesting as gradual cognitive decline, or due to disease-related pathology—manifesting as a more prominent cognitive decline. Irrespective of the pathology, the underlying brain structure and/or function are the conventional determinants of cognitive health. Complementing these determinants are the constructs of brain reserve (corresponding to structure) (Coffey et al., 1999) and cognitive reserve (corresponding to function) (Stern, 2002), whose presence (or lack thereof) could maintain (or negatively affect) cognitive performance. For example, older individuals could be (a) super agers, with high reserve and preserved brain structure and function, showing preserved cognition (de Godoy et al., 2020); (b) normal agers, with altered brain structure and function, showing gradual cognitive decline; or (c) poor agers, with low reserve and significantly altered brain structure and function and significant cognitive decline (Solé-Padullés et al., 2009). Altogether, a balance among structure, function and reserve most likely regulates an individual's ability to compensate in the face of pathology. In the present study, using functional MRI, we demonstrated the association of functional connectivity with the cognitive subdomain of verbal fluency and how cognitive reserve mediates this association, likely reflecting compensation in normal aging.

It must be stipulated that the construct of reserve has been described and reported predominantly in relation to broad measures of cognition, with greater emphasis on memory (Nyberg et al., 2012; Steffener and Stern, 2012; Opdebeeck et al., 2016). It is unclear whether distinct measures of cognition could be associated with specific compensatory mechanisms as it pertains to reserve (Stern et al., 2018b). Understanding of cognitive domain-specific compensation is important as not all cognitive domains evolve similarly with aging. Thus, in our current work, we focused specifically on verbal fluency, while future studies should investigate other language and non-language cognitive functions to further inform on universal vs. cognitive domain-specific compensation. In particular, we investigated the specific domain of phonemic fluency and tested the hypothesis that older individuals with high cognitive reserve can maintain phonemic fluency possibly by recruiting both linguistic and non-linguistic networks (Gonzalez-Burgos et al., 2020). In the current study, we validated this hypothesis by showing that greater functional connectivity of the Wernicke module with non-Wernicke structures (ipsilateral and contralateral cuneus, precuneus, calcarine cortex and lingual gyrus) was associated with higher performance in phonemic fluency. Further, this association was not mediated by cognitive reserve in the younger adults but was mediated by cognitive reserve in the older adults, thus, presumably representing compensation in the elderly. Our findings are relevant as verbal fluency is significantly impaired in several dementia types including Alzheimer's disease (Henry et al., 2004), shows early changes in mild cognitive impairment (Murphy et al., 2006), is sensitive to subjective cognitive decline (Nikolai et al., 2018), and is even suggested as a potential screening tool (McDonnell et al., 2020; Frankenberg et al., 2021). Further, subdomains of verbal fluency such as phonemic and semantic fluency evolve differentially over time, even in healthy aging (Stolwyk et al., 2015; Gonzalez-Burgos et al., 2019; Rodríguez-Aranda et al., 2020). Relative stability of phonemic fluency over aging, thus, raises the question of whether reserve-facilitated compensatory mechanisms might have a role in its reduced susceptibility to decline. Below we contextualize our findings, discuss the potential implications, and outline future directions.

We investigated an extended language network, beyond the classically identified Wernicke's and Broca's areas, by including the Wernicke's, Broca's, thalamic and anti-correlated modules (Tomasi and Volkow, 2012). Across these four modules, we observed four spatially distinct patterns of functional connectivity with involvement of frontal, temporal, parietal and subcortical brain regions, therefore extending beyond traditional Wernicke's and Broca's areas. This is consistent with the notion that language processing is supported by multiple and distributed sub-networks (modules) rather than individual specialized brain regions (Fedorenko and Thompson-Schill, 2014). Functional connectivity pattern in each of these modules also differed between age groups. On the one hand, we found involvement of additional brain regions in older adults relative to younger adults within the Wernicke and Broca modules. On the other hand, we found involvement of fewer brain regions in older adults relative to younger adults in the thalamic and anti-correlated modules (Figure 1). Such a differential pattern has often been attributed to aging (Logan et al., 2002; Cabeza and Dennis, 2012) and observed in other brain networks including visual (Park et al., 2004; Geerligs et al., 2014) and motor (Carp et al., 2011) networks. Irrespective of the module, we found that older adults had diminished functional connectivity compared to the younger adults (Figure 2). This implies that the older brains are more vulnerable to breakdown of within-module connectivity (Ferreira and Busatto, 2013; Sala-Llonch et al., 2015; Damoiseaux, 2017), thus, increasing the likelihood of need for compensation at older age.

Firstly, out of the four investigated functional modules, we identified that functional connectivity of the Wernicke module was a correlate of phonemic fluency in the combined cohort (Figure 3). However, this finding is incongruent with our hypothesis that functional connectivity involving the Broca's area (inferior frontal gyri) would be associated with phonemic fluency. The Broca's area, part of the Broca module in our study, has been implicated in language production (Meinzer et al., 2009). In contrast, the Wernicke's area (superior temporal gyri), part of the Wernicke module in this study, is traditionally known to be involved in language comprehension. This discordance could have three explanations: (a) the Wernicke module is broader than and extends beyond the Wernicke's area, comprising additionally temporal, parietal, and frontal brain regions. Particularly, the contribution of the (inferior, middle, superior) frontal regions has been implicated in phonemic fluency task activation, both in cognitively normal (Wagner et al., 2014) and aphasic patients (Perani et al., 2003); (b) even if the Wernicke's area may predispose the Wernicke module to be predominantly associated with semantic fluency (for semantic and lexical abilities), the module could still be important for phonemic fluency. Comparing semantic and phonemic fluency in cognitively normal individuals has shown a prominent separation between the semantic categories of animate and inanimate entities within phonemic fluency (Schwartz et al., 2003). Therefore, it is possible that phonemic fluency is not purely phonemic and has a pervasive semantic facilitation; (c) contrary to the traditional view, the role of Wernicke's area may indeed extend to language production, perhaps not in facilitating the motor movements required for production but rather in enabling the knowledge needed to articulate prior to production (Binder, 2015), attributing a similar role to the Wernicke module. Altogether, we suggest that in our current cohort of cognitively normal individuals, where all participants demonstrated normal levels of performance in phonemic fluency, variability in phonemic fluency is not primarily related to the normal functioning of Broca's area but on the efficient recruitment of non-Broca areas belonging to Wernicke and anti-correlated modules.

Secondly, we observed the functional connectivity of the Wernicke module with non-Wernicke regions to be correlated with phonemic fluency (Figure 3A). In fact, we observed engagement of regions from the anti-correlated module including both ipsilateral and contralateral cuneus, precuneus, calcarine cortex and lingual gyrus, which show a negatively correlated BOLD activity to that of the Wernicke module (Tomasi and Volkow, 2012). Cuneus, calcarine cortex and lingual gyrus are core to visual processing while precuneus is key for the default mode network, hence involved in attention and memory. This association between functional connectivity involving language-specific brain areas and phonemic fluency was not significant when adjusting for mental tracking and cognitive flexibility, suggesting that the variation in cognitive flexibility across age groups may moderate the association. This highlights the need to investigate the role of frontal regions such as the dorsolateral prefrontal cortex in relation to phonemic fluency in future studies. However, the association between functional connectivity and phonemic fluency persisted when adjusting for verbal memory and processing speed, validating that the observed association is specific to language processing to a certain degree. This implies that phonemic fluency elicited connectivity of language-specific task-positive (Wernicke module) and concomitantly task-negative (anti-correlated module) networks, suggesting the dichotomized functional organization in the brain (Fox et al., 2005). Overall, these findings underline the importance of investigating not only within-network but also outside-network connectivity.

Finally, our findings divulged cognitive reserve as a partial mediator of the relationship between functional connectivity (of Wernicke with anti-correlated module) and phonemic fluency in the combined cohort (Figure 4). Further investigation revealed that cognitive reserve is an unlikely mediator of phonemic fluency in younger adults while it is a partial mediator in older adults, thus, emphasizing the need for compensation at an older age. Notably, older adults with low cognitive reserve and low functional connectivity exhibited significantly lower phonemic fluency, whereas older adults with high cognitive reserve performed significantly better and comparable to the younger adults regardless of functional connectivity, thus, demonstrating the occurrence of compensation at an older age. Neuroanatomically, these effects were localized in the calcarine cortex, lingual gyrus and precuneus. The contributions of structures from the anti-correlated module (calcarine cortex, lingual gyrus, precuneus), have been shown to be part of the network topography expressing differential deactivation in aging (Stern et al., 2005). Given the involvement of Wernicke and the corresponding anti-correlated modules specific to phonemic fluency, future studies should examine correlates and compensation underlying other specific modalities of verbal fluency (semantic, action) and other language components or cognitive domains, to further understand whether the involvement of calcarine cortex, lingual gyrus and precuneus is specific to phonemic fluency, verbal fluency, or compensation of cognition in general.

This study has some limitations. As this is a cross-sectional study, we could not track longitudinal trajectories and changes in phonemic fluency and compensation. We included imaging data and corresponding phonemic fluency performance at the first available timepoint, which was not necessarily the baseline timepoint. This may have induced a learning effect in phonemic fluency that we tried to circumvent by accounting for potential learning effects in our statistical models. Further, we observed a significantly lower (although normal) global cognition (MMSE) in the older adults than in younger adults. The age effect on global cognition is a common finding in the aging literature (Tombaugh and McIntyre, 1992; Bravo and Hébert, 1997). Our result regarding age-related differences in phonemic fluency likely reflects an overall decline in cognition, as illustrated by the finding of non-significant age differences in phonemic fluency when controlling for MMSE. Hence, future studies in pathologies primarily affecting phonemic fluency are needed to further understand compensatory processes specific to pathology affecting phonemic fluency. As a proxy for cognitive reserve, we used the WAIS-III Information subtest, while there exist other proxies based on factors such as education, occupation, lifestyle, etc. However, the WAIS-III Information subtest was recently demonstrated to better capture premorbid ability, being superior to other proxies of cognitive reserve in the current cohort (Correia et al., 2015; Ferreira et al., 2016), and is also validated as an appropriate measure of reserve longitudinally (Elkana et al., 2019). Finally, in contrast to most studies on compensation using task-based functional MRI, we used resting-state MRI to derive the neural correlates of verbal fluency and investigate potential compensatory mechanisms. Although resting-state data would help us elucidate task-invariant networks, it may be limited in the specificity of accurately deriving the most relevant neural correlates of cognition functions.

In conclusion, we delineated that functional connectivity involving brain areas shared by the Wernicke (linguistic) and anti-correlated (non-linguistic) modules was associated with phonemic fluency in aging. This association was mediated by cognitive reserve in the older adults but not in the younger adults, indicating compensation for the effect of aging in phonemic fluency in the elderly. Extending our current analyses to other language and non-language cognitive functions is important and expected to provide a deeper understanding of universal versus domain-specific compensatory mechanisms.

## Data Availability Statement

The data analyzed in this study is subject to the following licenses/restrictions: The raw dataset comprising human neuroimaging data are part of the GENIC-database (Group of Neuropsychological Studies of the Canary Islands, University of La Laguna, Spain. Principal investigator: Professor JB) with identifiable information. The anonymized data may be available upon reasonable request by contacting the authors. Requests to access these datasets should be directed to DF, daniel.ferreira.padilla@ki.se.

## Ethics Statement

The studies involving human participants were reviewed and approved by Ethics committee of the University of La Laguna, Spain. The patients/participants provided their written informed consent to participate in this study.

## Author Contributions

RM: conceptualization, data preparation, data analysis, manuscript writing, and revision. LG-B: conceptualization, data collection, data preparation, data analysis, manuscript writing, and revision. LD-F: data curation and manuscript revision. J-SM: data preparation and data processing. JB: data collection, manuscript revision, and funding. DF: conceptualization, data collection, supervision, and manuscript revision. EW: supervision, manuscript revision, and funding. All authors have read the approved the submitted manuscript.

## Conflict of Interest

The authors declare that the research was conducted in the absence of any commercial or financial relationships that could be construed as a potential conflict of interest.
