# Supplementary material for: Functional Connectivity and Compensation of Phonemic Fluency in Aging
Source: Front Aging Neurosci. 2021 Jul 5;13:644611. doi: 10.3389/fnagi.2021.644611 (PMC8287584; doi:10.3389/fnagi.2021.644611)
Supplement: Supplementary file 1 [file Data_Sheet_1.PDF]

Functional Connectivity and Compensation of Phonemic Fluency in Aging

Supplementary Material

Supplementary Figure 1. Organization of the four modules in the extended language network

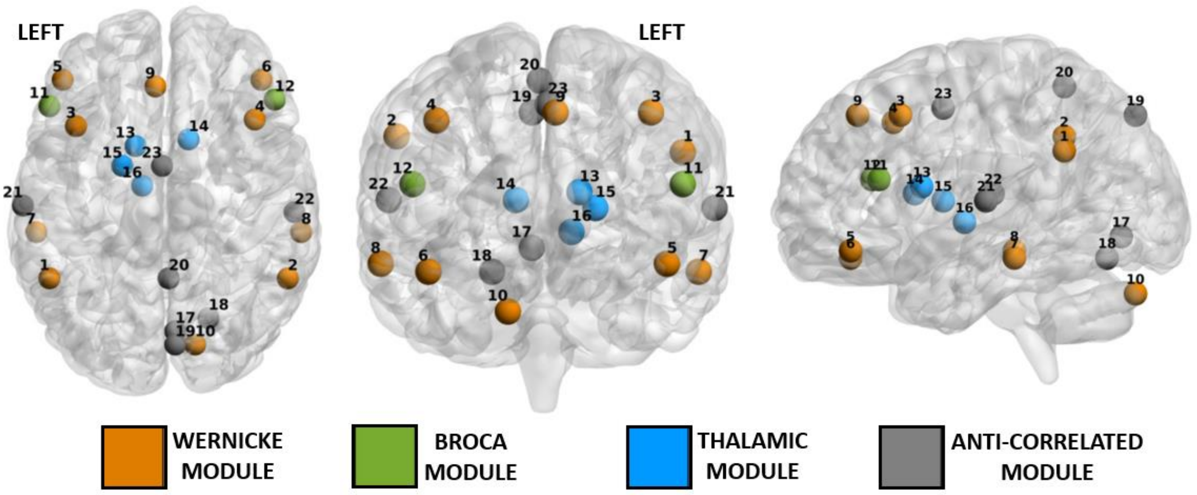

Labels corresponding to the indices of the brain regions can be found listed in Supplementary Table 1. The visualizations were generated with BrainNet Viewer (Xia, Mingrui, Jinhui Wang, and Yong He. "BrainNet Viewer: a network visualization tool for human brain connectomics." *PloS one* 8, no. 7 (2013): e68910).

Supplementary Table 1. Spatial coordinates of the extended language network

Index	Brain region	Brodmann Area	MNI x (mm)	MNI y (mm)	MNI z (mm)
**Wernicke Module**					
1	Wernicke's area	39/40	-51	-51	30
2	Right inferior parietal	40	57	-51	36
3	Middle frontal	46	-39	18	45
4	Pars opercularis	44	42	21	42
5	Left pars orbitalis	47	-45	39	-12
6	Right pars orbitalis	47	45	39	-15
7	Left inferior temporal	21/20	-57	-30	-15
8	Right inferior temporal	21/20	63	-30	-12
9	Superior frontal	8	-3	36	45
10	Cerebellum	crus	15	-81	-30
**Broca Module**					
11	Broca's area	45	-51	27	18
12	Pars triangularis	45	51	30	18
**Thalamic Module**					
13	Left caudate	-	-12	9	15
14	Right caudate	-	12	12	12
15	Putamen/globus pallidus	-	-18	0	9
16	Ventral thalamus	-	-9	-9	0
**Anti-correlated Module**					
17	Striate	17	6	-75	-6
18	Extrastriate	18	21	-69	-15
19	Posterior parietal	7	6	-81	45
20	Superior Parietal	5	3	-51	57
21	Left superior temporal	42	-63	-18	9
22	Right superior temporal	42	60	-21	12
23	Cingulate	24	0	0	48
